# Negative-Pressure Wound Therapy for the Management of Complex Surgical Wounds in a Minority Population

**DOI:** 10.7759/cureus.56726

**Published:** 2024-03-22

**Authors:** Max Murray-Ramcharan, Michelle Feltes Escurra, Ryan Engdahl, Federico L Gattorno

**Affiliations:** 1 General Surgery, Harlem Hospital Center, Harlem, USA; 2 Plastic Surgery, Harlem Hospital Center, Harlem, USA; 3 General Surgery, Woodhull Medical Center, Brooklyn, USA

**Keywords:** delayed wound healing, incisional negative pressure wound therapy, necrotizing soft tissue infection (nsti), negative-pressure wound therapy, negative pressure device

## Abstract

Introduction

Negative-pressure wound therapy (NPWT) has been used for decades as an established treatment modality for complex wounds, now commonplace in hospitals and various clinical and outpatient settings. Several studies have noted improved healing outcomes with this device, but the current state of literature is in debate on both clinical and economic effectiveness. The use of NPWT can become expensive, largely because of the complexity of wounds and the need for outpatient management, from which a majority of the benefit is derived. This creates a disparity in access to this therapy. A lack of insurance and limited access to healthcare that is present in minority populations contribute to this inequality.

Methods

We reviewed the clinical courses of eight patients who were treated with NPWT at a single acute care facility in an underserved area caring for a minority population.

Results

We describe several different anatomic wounds along with details including the size of wounds, number of debridements, length of hospital stay, duration of treatment, and ensuing courses of the minority patients who received NPWT for the entire duration of their wound care course.

Conclusions

This case series demonstrates desirable wound healing outcomes with the use of NPWT in the minority population. The authors draw attention to the outpatient benefit of this device that may be lost in those with limited insurance in minority populations and seek to encourage further studies in this population in resource-limited settings to determine its true clinical effectiveness.

## Introduction

Negative-pressure wound therapy (NPWT), by way of a vacuum-assisted closure (VAC, KCI Concepts, San Antonio, Texas) device, was introduced to clinical practice over two decades ago as an adjunct therapy for many types of complex wounds. Its effectiveness has since been debated in many studies, with large randomized controlled trials (RCTs) noting non-inferiority to standard wound care [1], and large meta-analyses noting improved outcomes in wound healing [2,3]. These results, along with smaller institutional studies and provider experience, have led to this wound care method being extensively integrated into practice by surgeons, physicians, and wound care specialists, with the goal of enhanced wound healing [4]. Multiple cost-effectiveness models have demonstrated the advantages of NPWT in complex wounds by promoting shorter hospital durations and quicker return to work [5,6]; however, several analyses contradict these results, advocating no significant cost benefit. Regardless, because of device costs and the need for insurance for use, patients lacking insurance or those in minority populations are not always able to benefit from NPWT, especially in the outpatient setting. Because of these barriers, NPWT may be forgone in this population with outpatient dressings conducted with standard wound care instead. The authors present the clinical courses of eight such patients with the aim of demonstrating the clinical effectiveness of NPWT in the minority population.

## Materials and methods

We present the following eight (n=8) cases of complex wounds of varied etiologies and in different anatomic locations within a minority population to demonstrate the range and clinical effectiveness of NPWT. In all cases, the nontreated black sponges were used and sized individually for each patient to incorporate the entire wound. The cases are summarized in Table 1.

**Table 1:** Summary of patients in case series NPWT – Negative-Pressure Wound Therapy; T2DM – Type 2 Diabetes Mellitus; CKD – Chronic Kidney Disease; RA – Rheumatoid Arthritis; AA – African American; HTN – Hypertension; CHF – Congestive Heart Failure; NSTI – Necrotizing Soft Tissue Infection; HCV – Hepatitis C Virus, PSA – Polysubstance Abuse

Patient demographics	Comorbidities	Wound type	Location of wound	Number of surgical procedures	Duration of NPWT (days)	Time until wound healed (days)	Approximate hospital length of stay (days)	Skin graft needed (Y/N)
Patient 1: 67-year-old Hispanic female	T2DM, CKD3, RA	Abscess	Right medial thigh	5	16	19	31	N
Patient 2: 60-year-old AA male	HTN, CHF, CKD	NSTI	Dorsum of foot and right leg	6	28	36	86	Y
Patient 3: 50-year-old Hispanic male	T2DM	NSTI	Right anterior upper leg	4	35	59	60	Y
Patient 4: 57-year-old AA male	T2DM, CKD5, HTN, Chronic anemia	Ulcer with acute or chronic osteomyelitis	Left lower extremity/plantar wound	2	36	45	59	Y
Patient 5: 65-year-old Hispanic male	T2DM, HCV, Alcohol use disorder	NSTI	Left lower extremity/left medial foot	2	43	56	56	N
Patient 6: 65-year-old Hispanic female	T2DM, Peripheral neuropathy	NSTI	Left anterior shin and dorsal foot	5	23	36	40	Y
Patient 7: 61-year-old AA male	PSA, Depression, HCV	NSTI	The posterior aspect of the left leg	8	30	33	33	Y
Patient 8: 52-year-old Hispanic male	T2DM, HTN	Abscess	Abdomen/periumbilical	2	30	33	43	N

## Results

**Figure 1:**
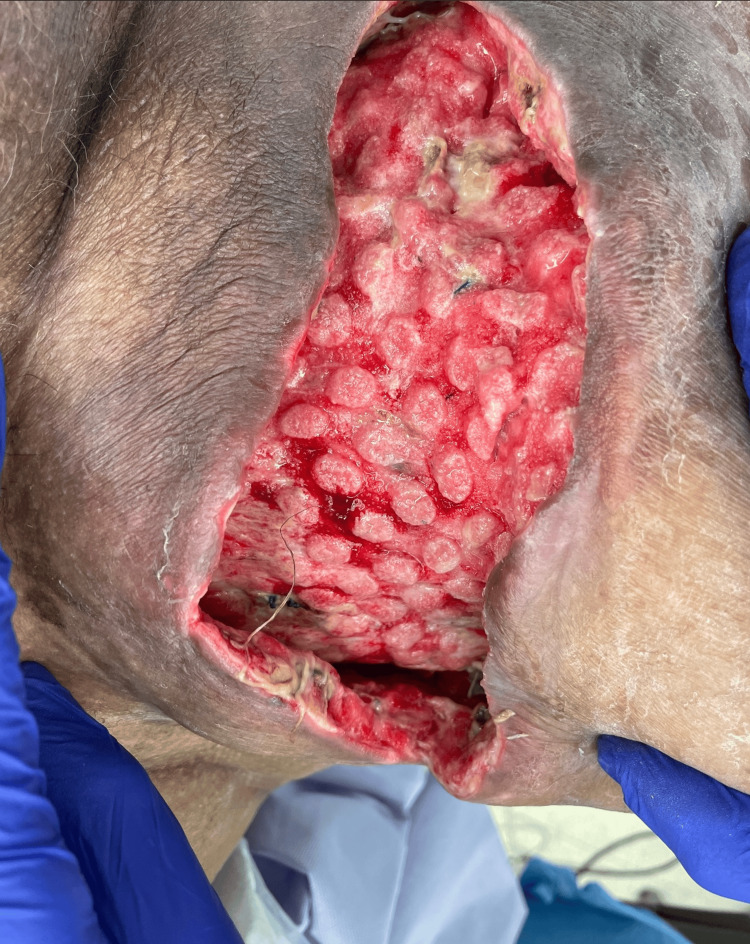
Right medial thigh wound after NPWT (Case 1) NPWT – Negative-Pressure Wound Therapy

Case 1: This patient is a 67-year-old female with poorly controlled type 2 diabetes mellitus (T2DM) and chronic kidney disease (CKD) who presented with a right upper medial thigh necrotizing soft tissue infection (NSTI) associated with pain, fluctuance, and purulent drainage. The patient underwent extensive incision and debridement of necrotic tissue with a 15 cm-long wound from the upper anterior thigh to the medial aspect. During her hospital course, the patient underwent four additional debridement procedures and, in the interim, received wound care with NPWT in various settings (Figure 1) including Veraflo (KCI Concepts, Acelity, San Antonio, TX) and regular VAC until discharge with portable VAC.

**Figure 2:**
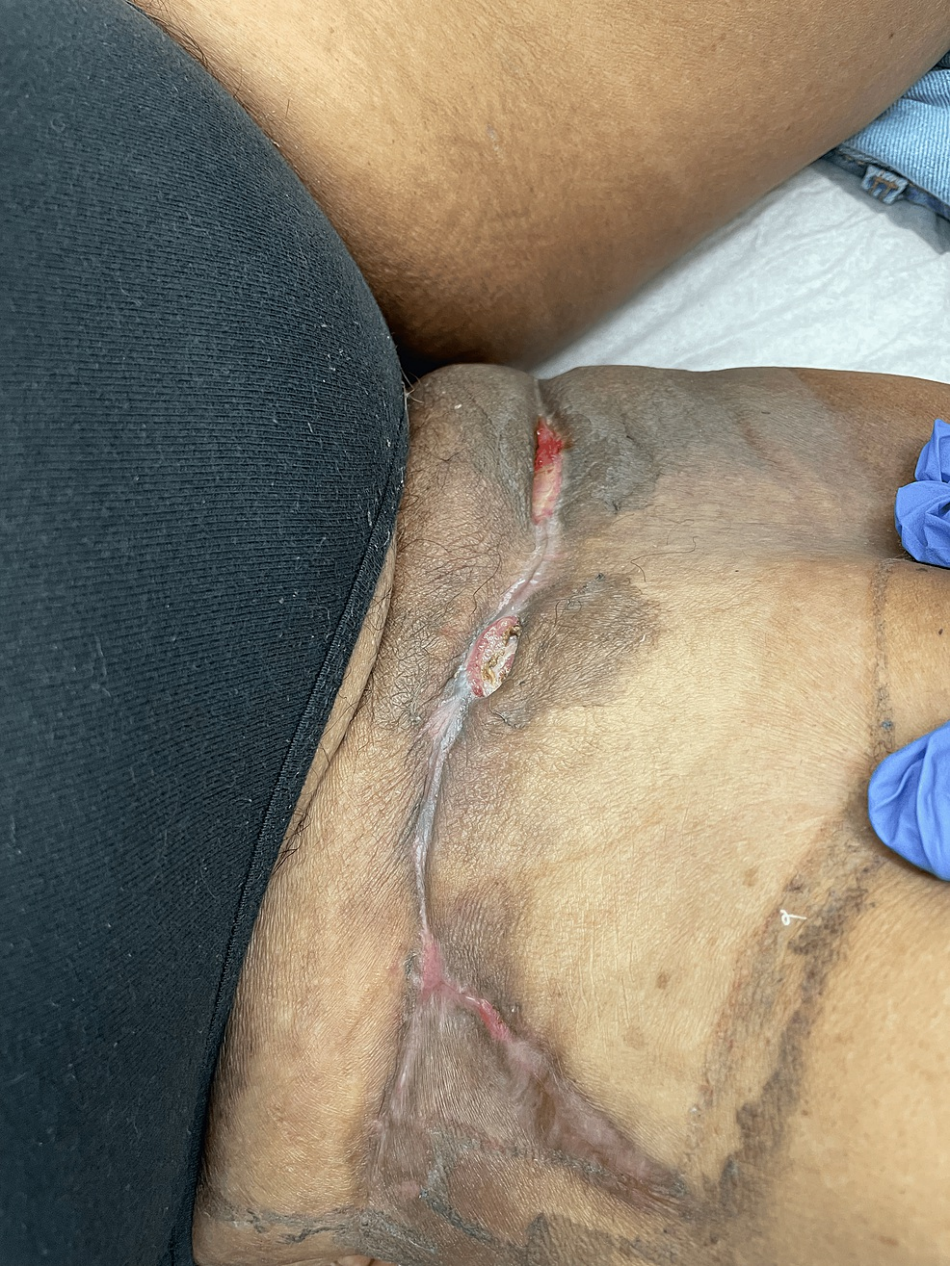
Healed right medial thigh wound (Case 1)

The patient had consistent follow-up visits to the clinic over the following six months with excellent cosmetic results (Figure 2).

**Figure 3:**
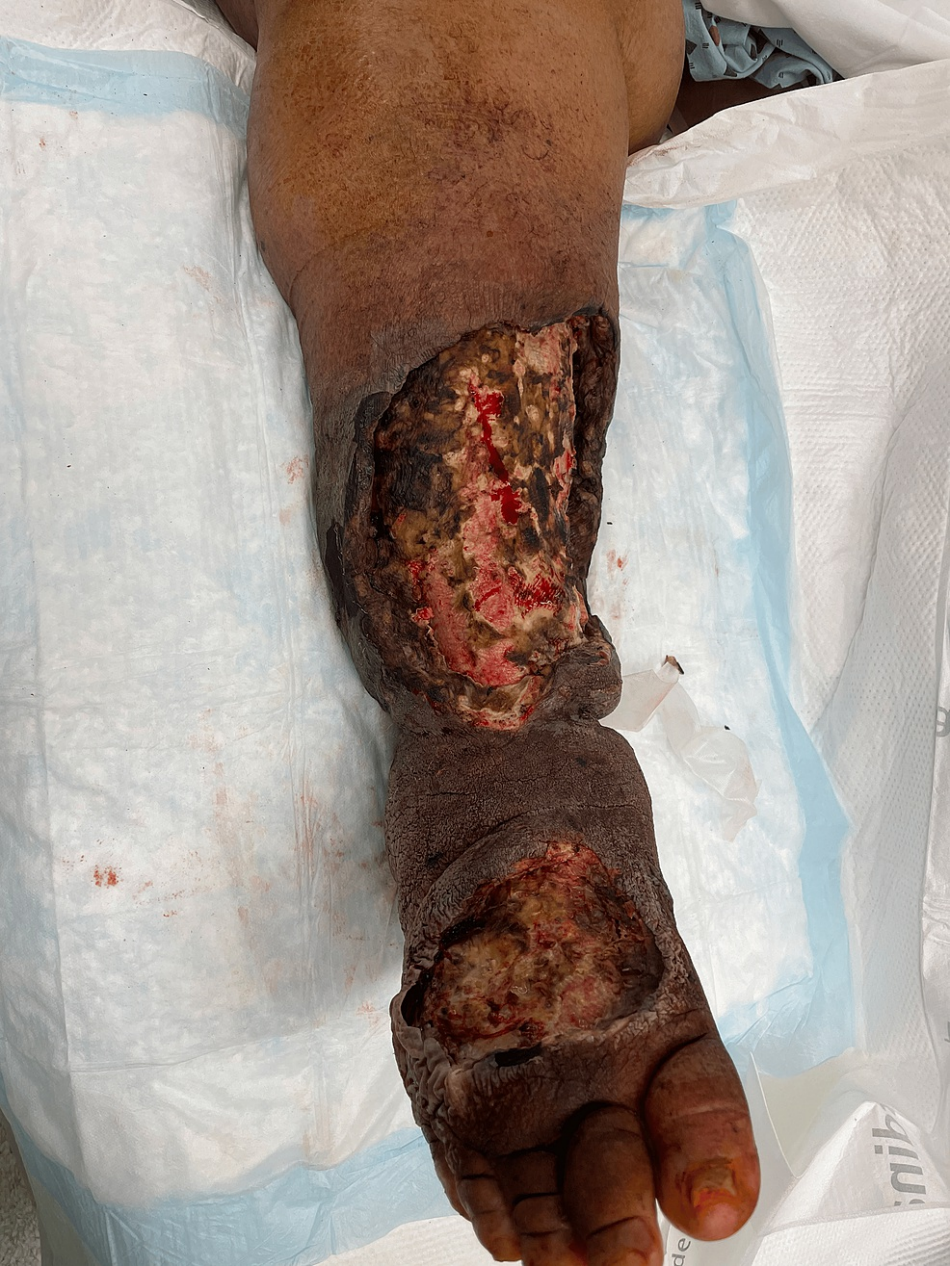
Right lower extremity wound after initial debridement (Case 2)

Case 2: This patient is a 60-year-old male with congestive heart failure (CHF), hypertension (HTN), and CKD who presented with altered mental status, hypotension, and bullae along the right lower extremity (RLE) along the anterior leg and dorsum of the foot associated with crepitus and elevated WBCs. The patient underwent emergent debridement of NSTI (Figure 3).

**Figure 4:**
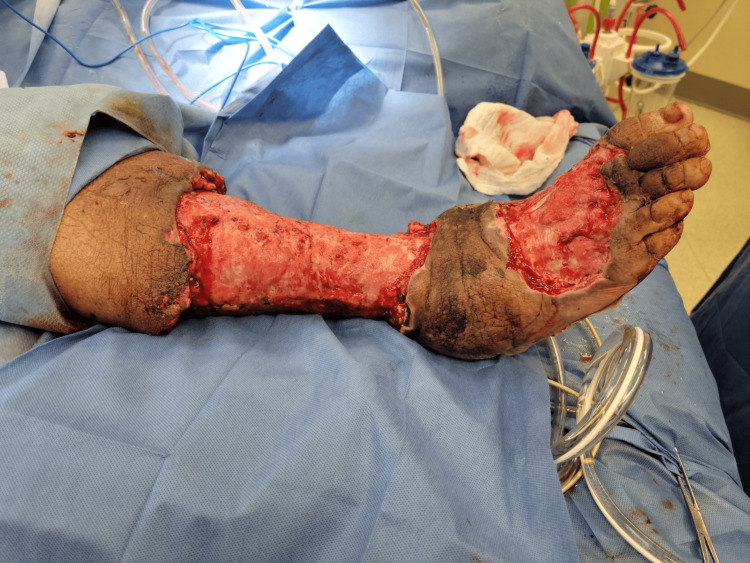
Right lower extremity wound after serial debridements (Case 2)

During his hospital course, the patient underwent five additional debridement procedures, and the wound was managed with NPWT (Figure 4).

**Figure 5:**
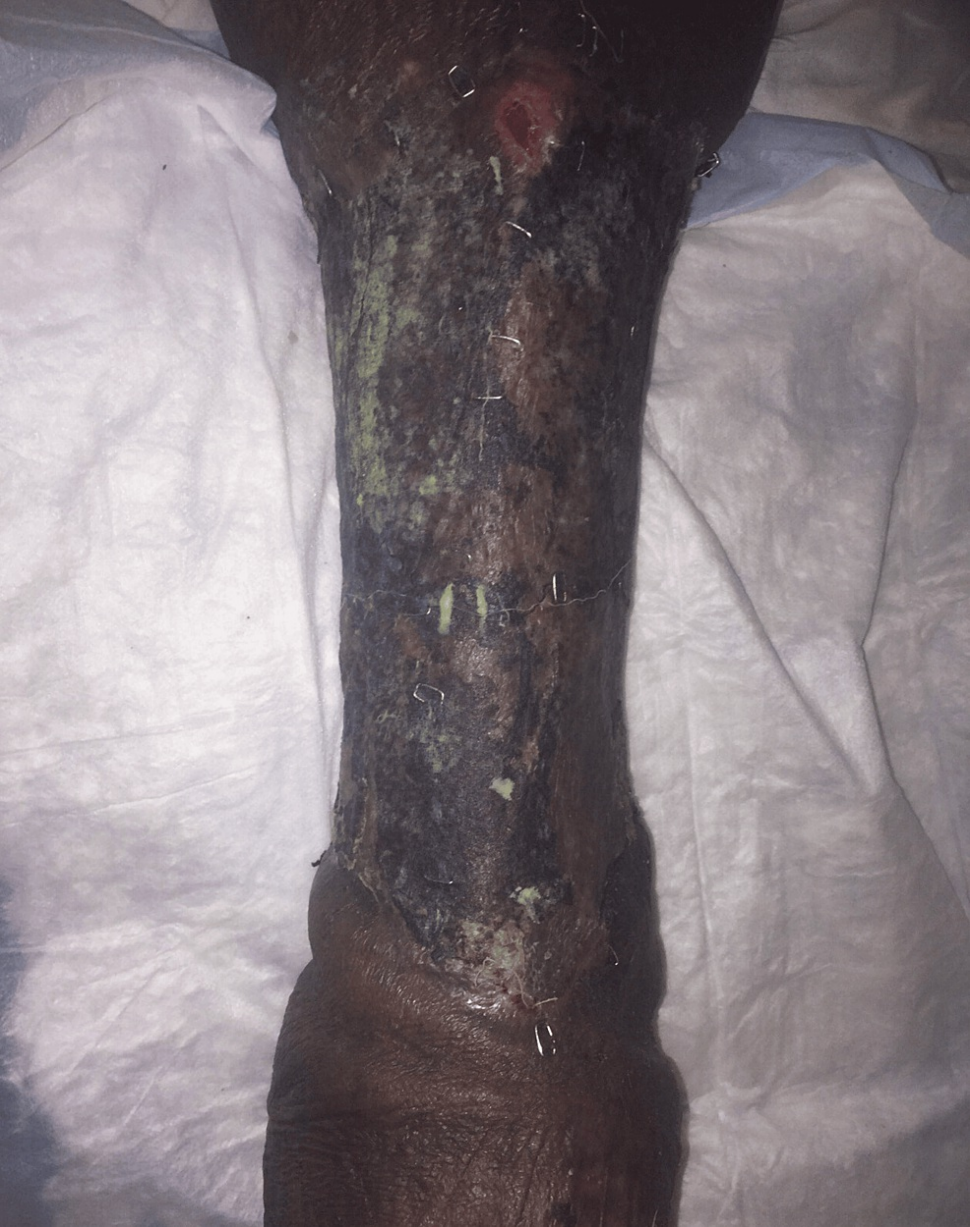
Healed right lower extremity wound after STSG (Case 2) STSG – Split-Thickness Skin Graft

**Figure 6:**
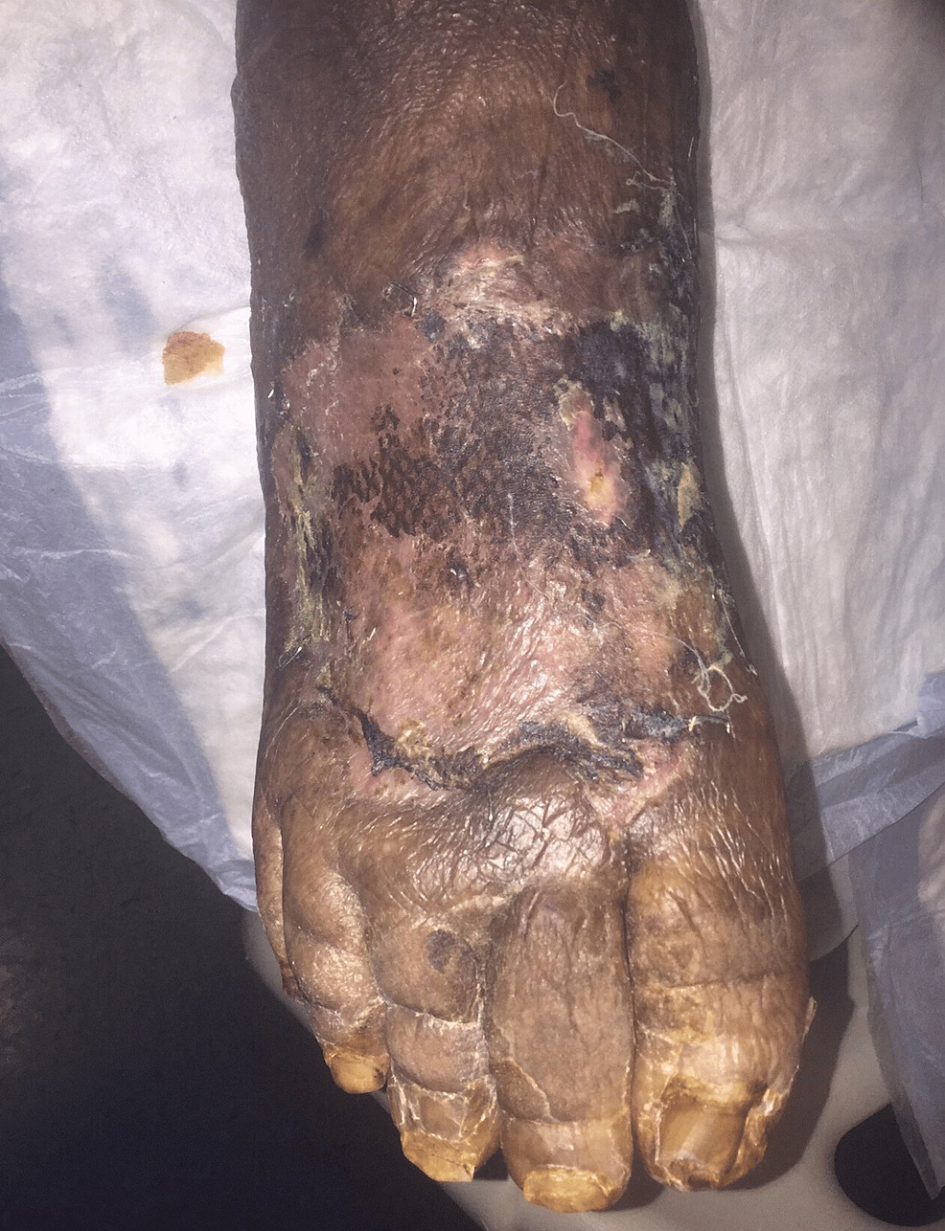
Healed right lower extremity foot wound after STSG (Case 2) STSG – Split-Thickness Skin Graft

After approximately three weeks of treatment, the patient underwent split-thickness skin graft (STSG), after which he remained inpatient for two weeks of local wound care, with a prolonged hospital course because of nonsurgical-related issues. He demonstrated desirable wound-healing results (Figures 5, 6).

**Figure 7:**
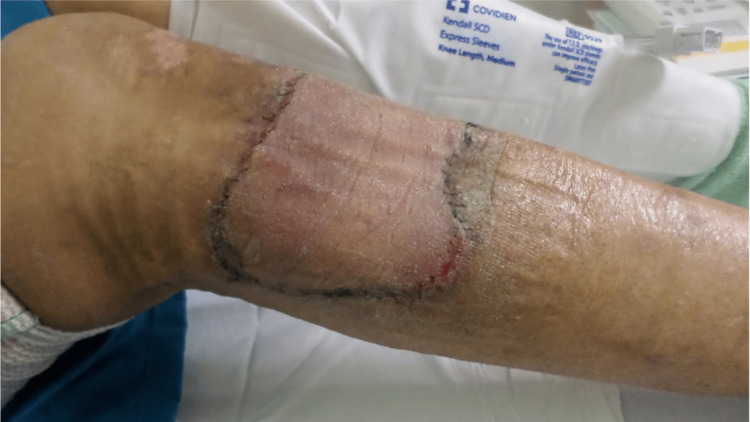
Right anterior leg after STSG (Case 3) STSG – Split-Thickness Skin Graft

Case 3: This patient is a 50-year-old male with poorly controlled T2DM who presented after a week of RLE pain and swelling after an injury to his anterior upper leg. A physical exam revealed severe cellulitis and induration of the RLE from the distal third of the thigh to the proximal third of the leg, along with a 10x8cm eschar with central fluctuance and palpable crepitus. This patient underwent emergent operative debridement of the RLE NSTI. During his hospital course, the patient underwent approximately three additional debridement procedures and was managed with NPWT on VeraFlo and regular VAC settings. After approximately five weeks of inpatient wound care with NPWT, he underwent STSG (Figure 7). After discharge, the patient consistently demonstrated excellent cosmetic results over a six-month follow-up period.

**Figure 8:**
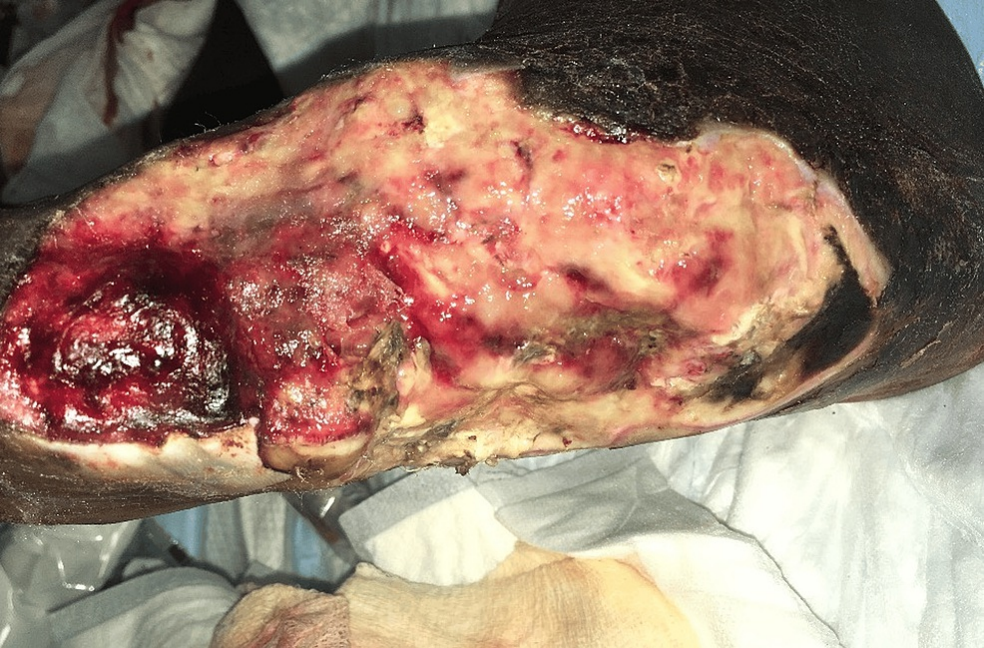
Left medial foot wound (Case 4)

Case 4: This patient is a 57-year-old male with a history of CKD, poorly controlled T2DM, HTN, and prior left transmetatarsal amputation (TMA) for the diabetic foot, presented with an infected necrotic ulcer over the existing TMA stump and medial foot. The patient underwent multiple debridements, with eventual, wound matrix, and VAC application (Figure 8).

**Figure 9:**
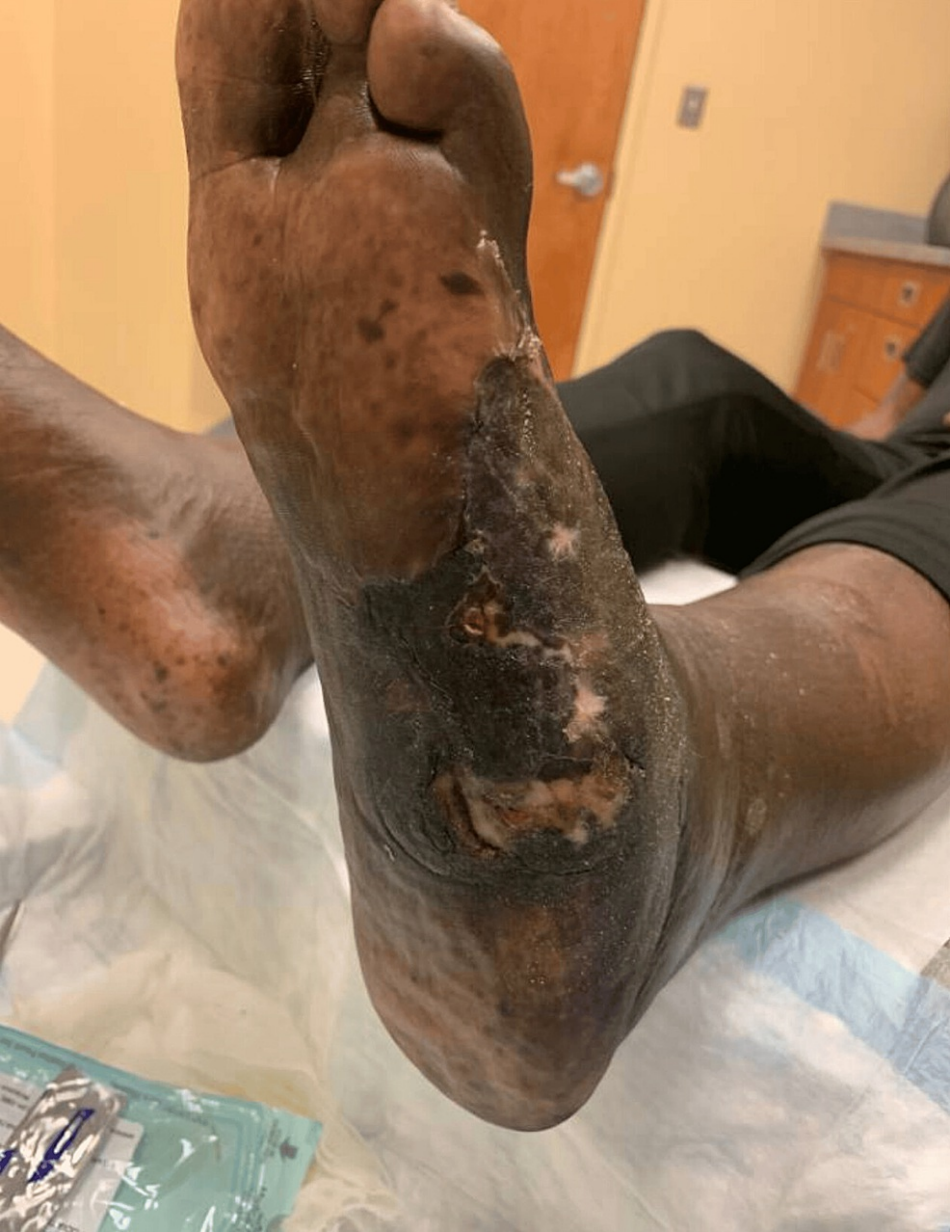
Healed left medial foot wound after STSG (Case 4) STSG – Split-Thickness Skin Graft

The patient continued with NPWT for three weeks, after which he underwent STSG. The patient remained in the hospital for social reasons for two additional weeks and received local wound care. He was followed-up regularly in the clinic and demonstrated great cosmetic results (Figure 9).

**Figure 10:**
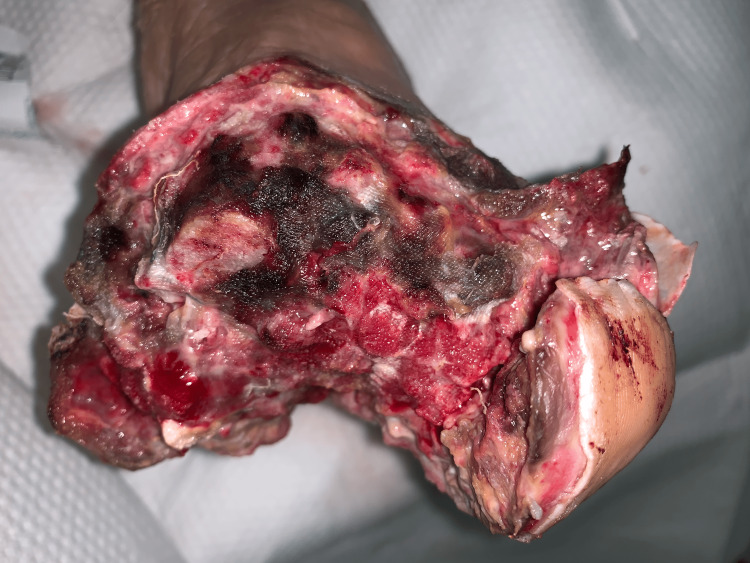
Left TMA wound after initial debridement (Case 5) TMA: Transmetatarsal Amputation

**Figure 11:**
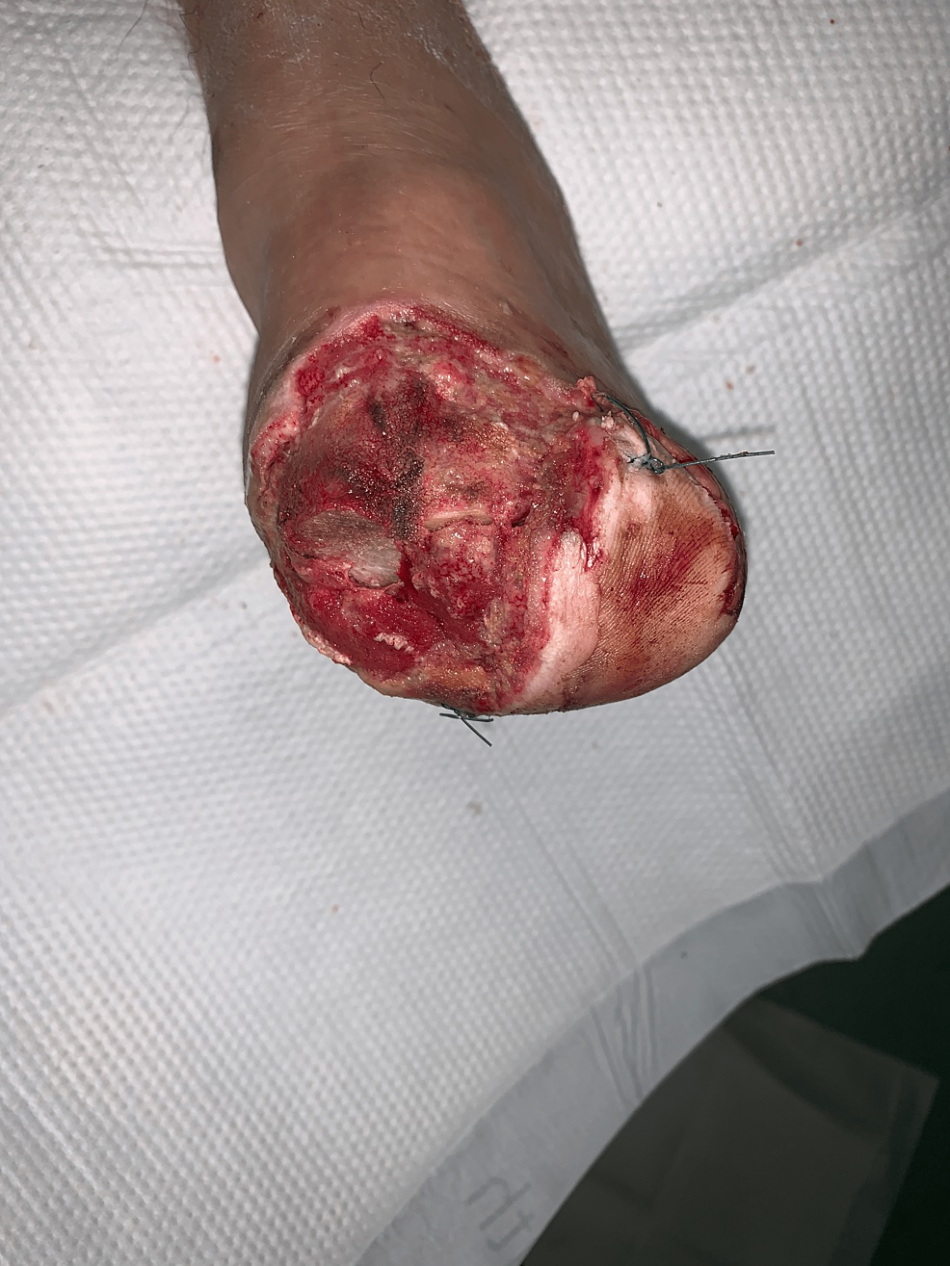
Left TMA wound after NPWT (Case 5) TMA – Transmetatarsal Amputation, NPWT – Negative-Pressure Wound Therapy

Case 5: This patient is a 65-year-old male with a history of poorly controlled T2DM, hepatitis C virus (HCV), and alcohol abuse and presented with left foot pain, chills, and subjective fevers. He was tachycardic and febrile upon presentation with elevated WBCs. Physical exam was notable for a plantar ulcer on his left foot with purulent drainage, cellulitis, and discoloration involving the first to third digits and crepitus along the dorsum of the foot extending up to the ipsilateral knee. The patient underwent urgent debridement for NSTI (Figure 10), with the ensuing hospital course notable for multiple additional debridement procedures, left TMA, and NPWT application after control of infection (Figure 11).

**Figure 12:**
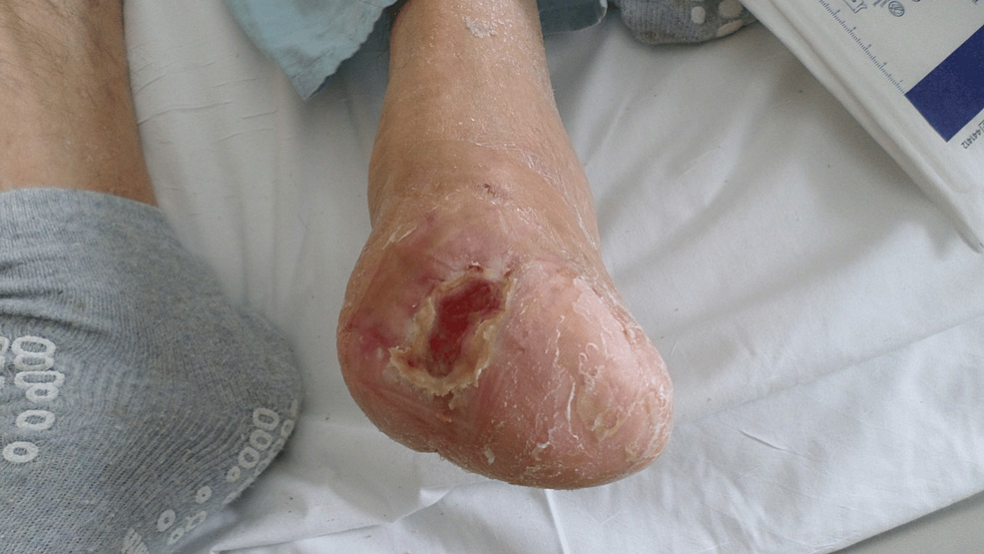
Healed left TMA wound (Case 5) TMA – Transmetatarsal Amputation

A combination of VeraFlo and regular VAC settings was used for two weeks, followed by local wound care until discharge. Outpatient follow-up was notable for desirable cosmetic results (Figure 12).

**Figure 13:**
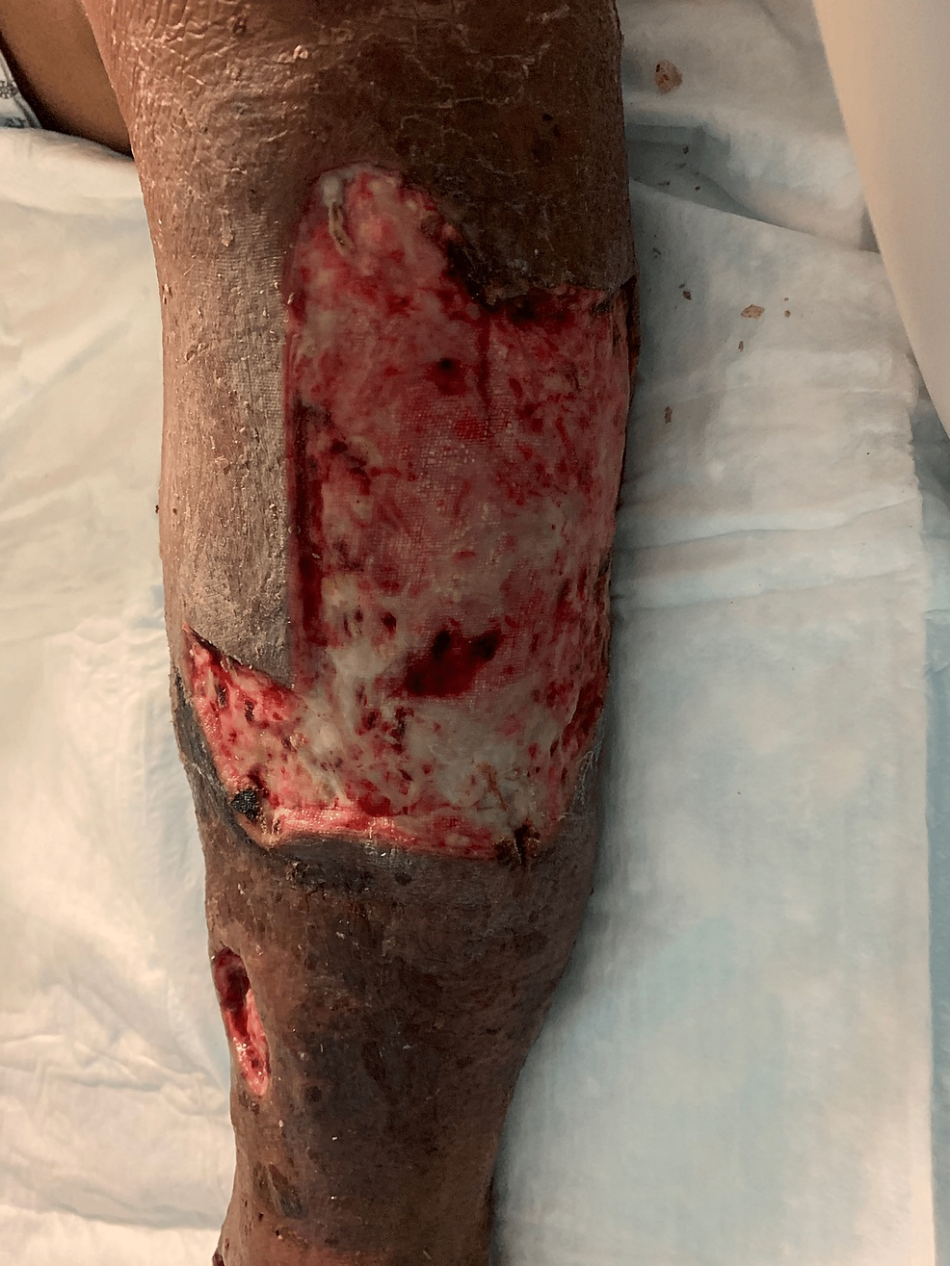
Left leg wounds after initial debridement (Case 6)

Case 6: This patient is a 65-year-old female with a history of T2DM with peripheral neuropathy, admitted for sepsis from left lower extremity (LLE) cellulitis. After failure to progress on antibiotics, a surgical evaluation revealed severe cellulitis with multiple abscesses along the LLE, and the patient underwent multiple bedside incision and drainage procedures, followed by formal washout and debridement (Figure 13) with placement of VAC on the left anterior shin as well as the dorsal foot. 

**Figure 14:**
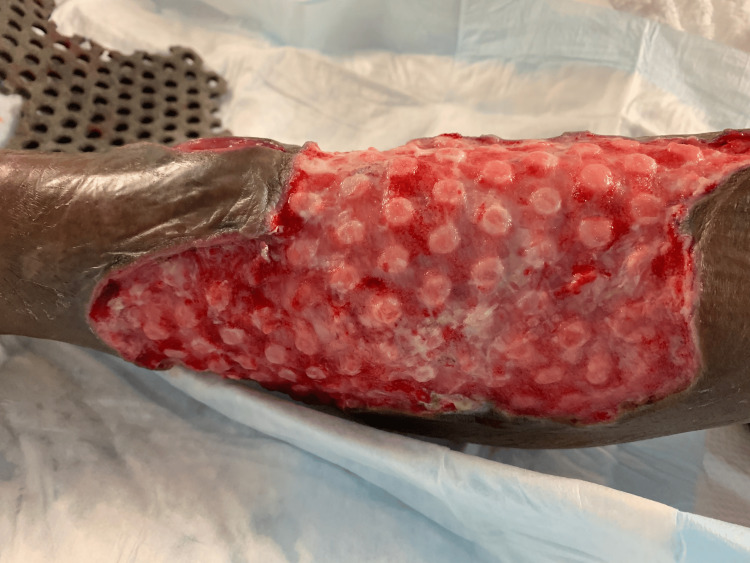
Left leg wounds after NPWT (Case 6) NPWT – Negative-Pressure Wound Therapy

The patient developed additional abscesses necessitating two additional debridements, with further VAC placement (Figure 14).

**Figure 15:**
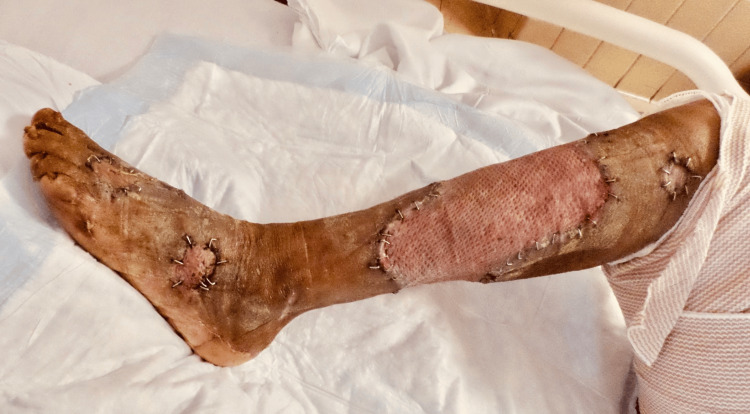
Left leg wounds after STSG (Case 6) STSG – Split-Thickness Skin Graft

After the wounds demonstrated good granulation tissue, the patient underwent matrix placement, followed by STSG of the LLE (Figure 15) and was discharged. 

**Figure 16:**
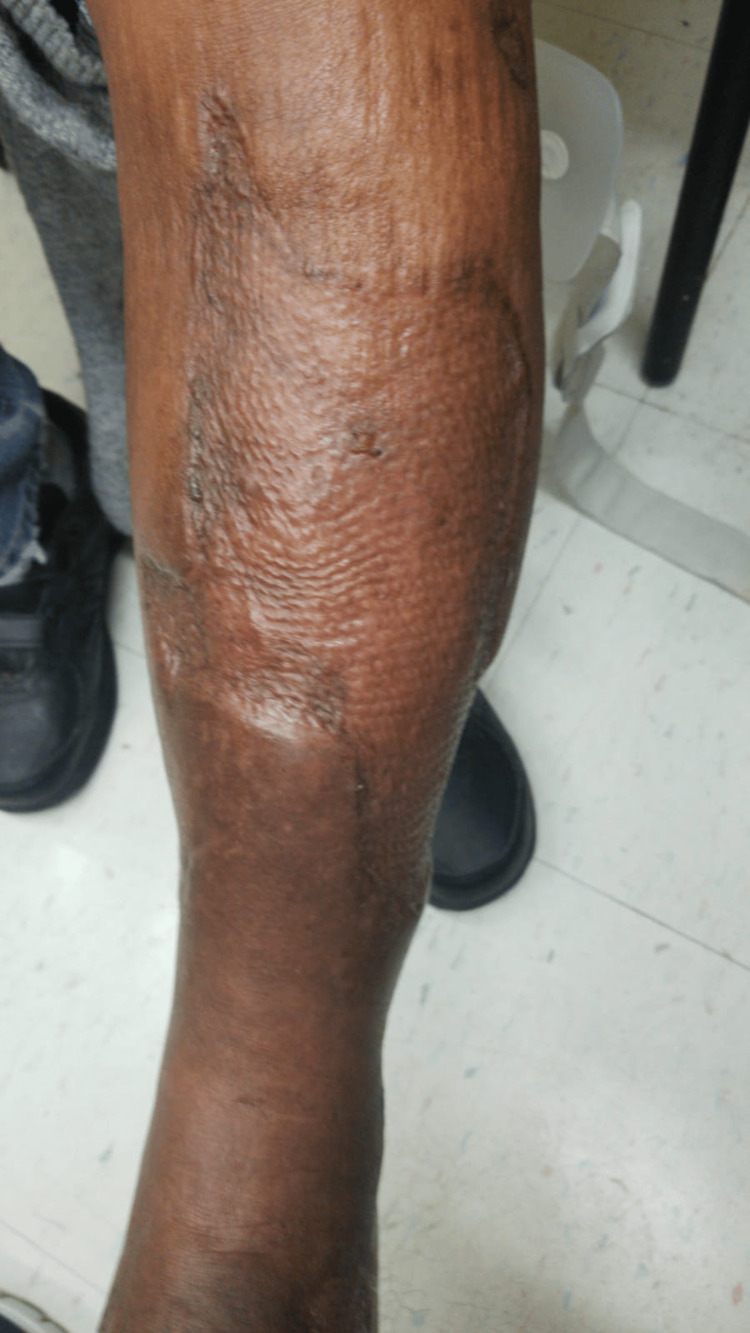
Healed left leg wounds (Case 6)

Following regular outpatient care for the next three months, the patient showed excellent cosmetic outcomes (Figure 16).

**Figure 17:**
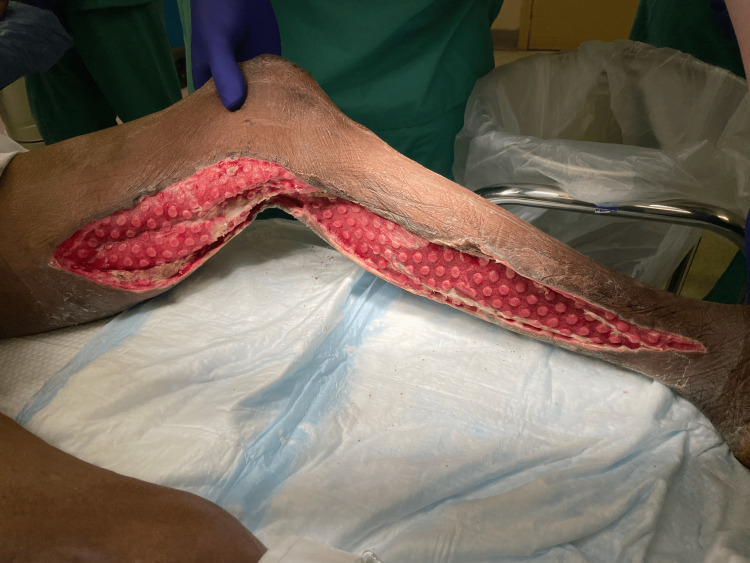
Left medial lower extremity wound after NPWT (Case 7) NPWT – Negative-Pressure Wound Therapy

Case 7: This patient is a 61-year-old male with a history of substance abuse and HCV who presented with medial left leg swelling, pain, cellulitis, warmth, and tenderness to palpation with associated crepitus. He had elevated WBCs and was hypotensive. The patient emergently underwent incision and debridement with intraoperative findings of NSTI. During his hospital stay, the patient underwent an additional six debridements and was later managed with NPWT including VeraFlo and regular VAC settings (Figure 17).

**Figure 18:**
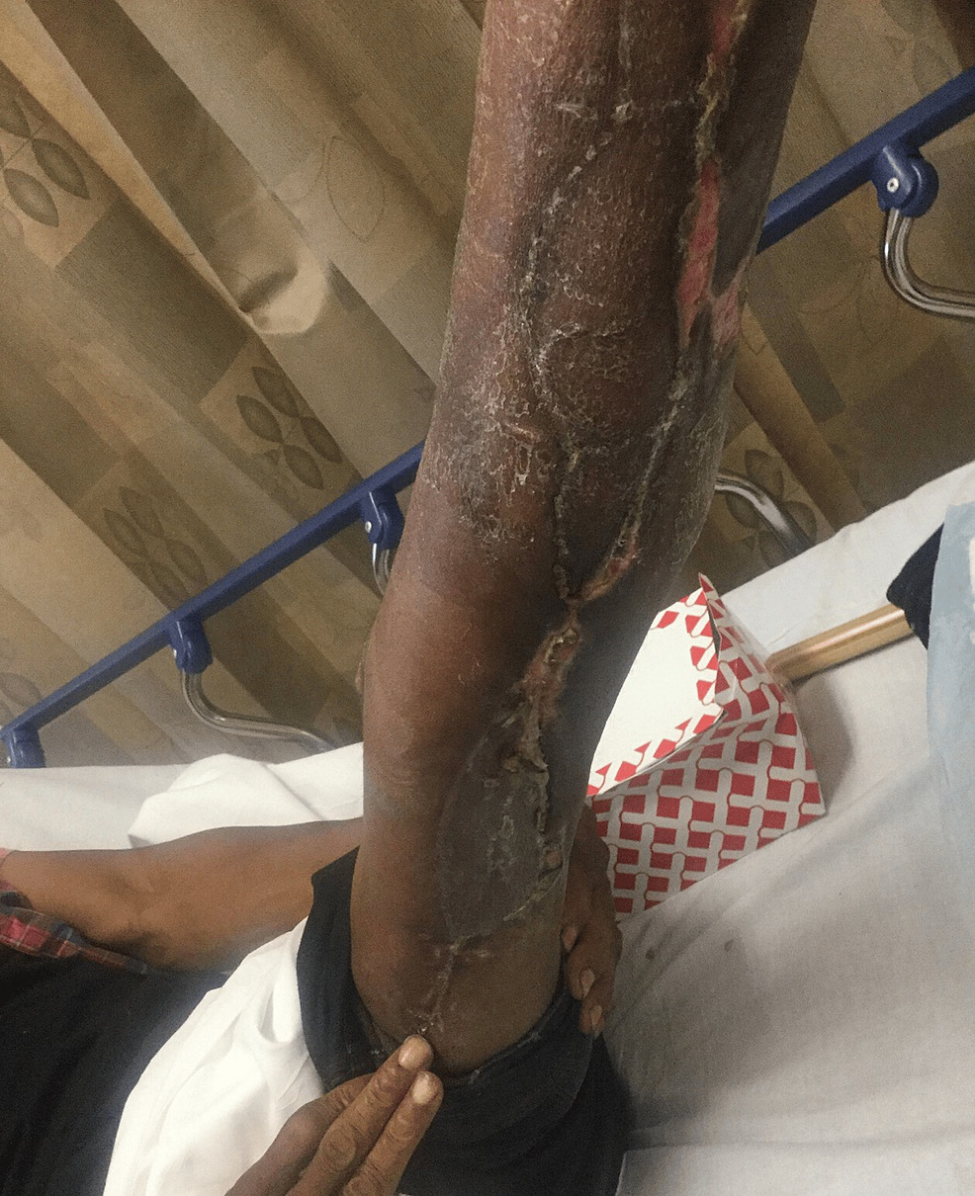
Healed left medial lower extremity wound after STSG (Case 7) STSG – Split-Thickness Skin Graft

After two weeks of NPWT, the patient underwent STSG and stayed an additional two weeks for local wound care. Follow-up outpatient was notable for great cosmetic results (Figure 18).

**Figure 19:**
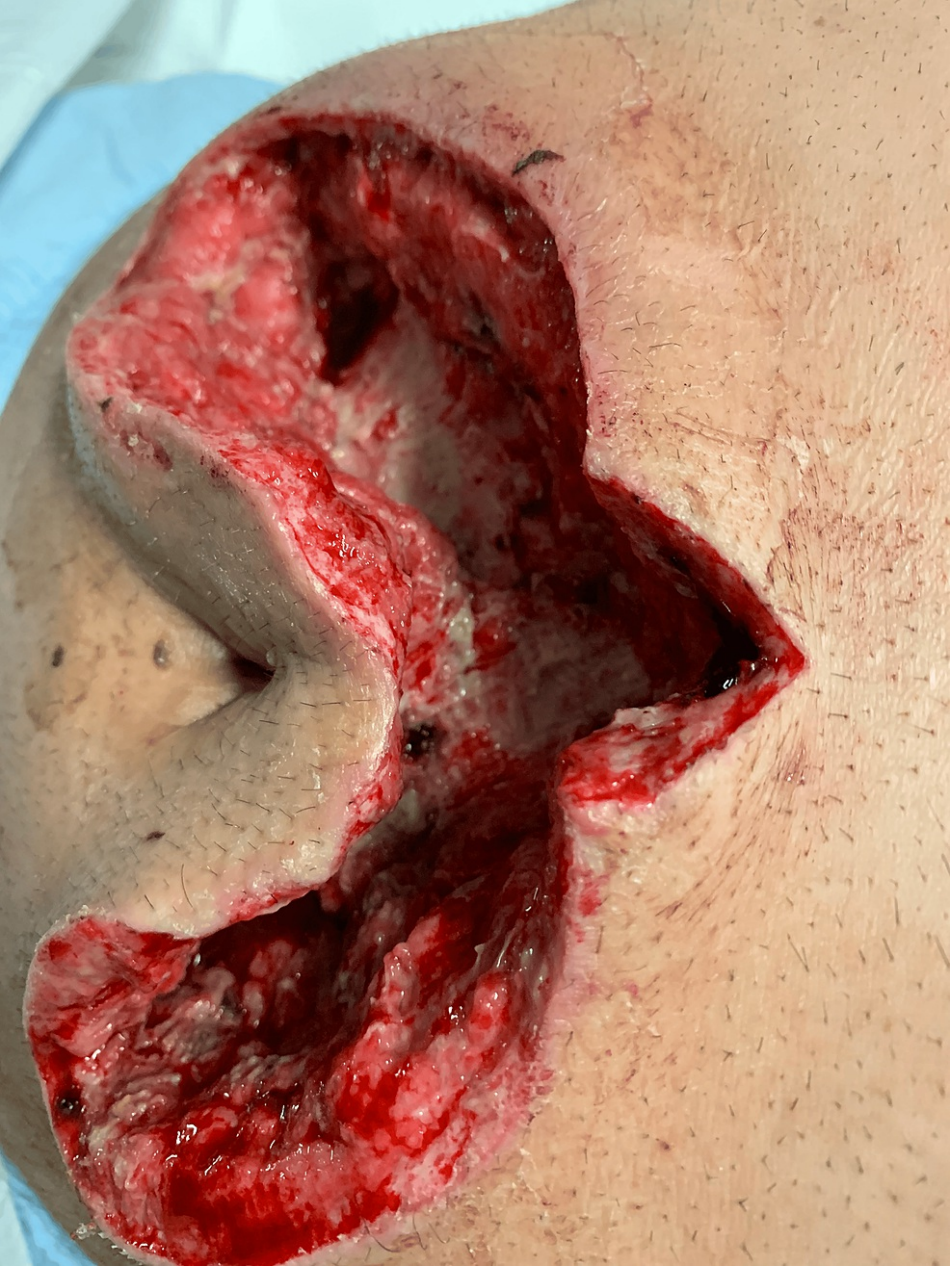
Abdominal wall wound after debridement (Case 8)

Case 8: This patient is a 52-year-old male with poorly controlled T2DM who presented with periumbilical pain, swelling, and erythema at his regular insulin injection site. He was febrile on admission and had extensive anterior abdominal wall erythema, induration, and tenderness on examination. His WBCs were significantly elevated, and an initial trial of antibiotics was unsuccessful, with worsening pain and the development of a large anterior abdominal wall abscess. The patient underwent wide debridement of NSTI, followed by another debridement a few days later (Figure 19).

**Figure 20:**
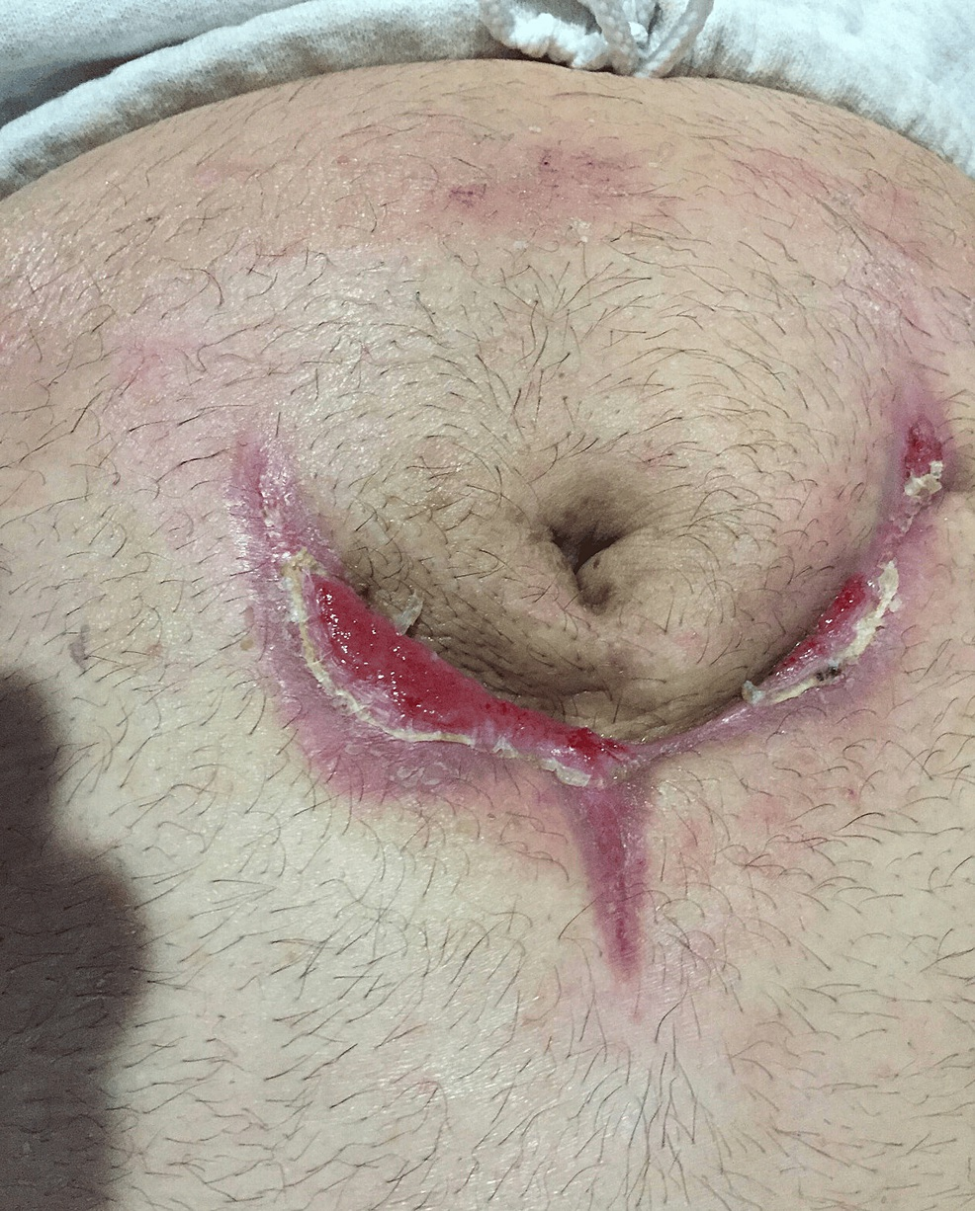
Healed abdominal wall wound (Case 8)

After infection control, the patient was managed with NPWT on Veroflo, shortly followed by regular VAC settings. On discharge, the wound was smaller and well granulated, with improved cosmesis and no residual infection (Figure 20).

## Discussion

Studies have described the economic burden of patients requiring complex wound care, and several cost-effectiveness analyses have been conducted to establish the benefits of various treatment options. In Trueman et al. [7], the authors not only conclude that NPWT provides economic benefit to standard wound care but also that this therapy may further unburden hospital-based care, leading to additional cost benefit. Vuerstaek et al., in an RCT published in the Journal of Vascular Surgery (2006) [8], the authors determined that NPWT in their study promoted shorter duration to healing, significantly lower total costs, increased quality of life (QOL), and decreased pain when compared to conventional therapy. These results have been echoed by numerous studies, with large meta-analyses concluding that these clear benefits should stimulate healthcare initiatives to invest in the provision of outpatient NPWT services to improve overall outcomes and economic burden [9]. However, more recent data from the Cochrane Database of Systematic Reviews [10] suggest that the superiority of current wound care properties and cost-effectiveness of NPWT is uncertain within the literature, leaving room for additional studies to determine true clinical effectiveness.

Trueman et al. [7] note in their analysis that a major shortcoming of NPWT is that many patients may be denied home care. This is observed mostly in the uninsured and low socioeconomic status patients and thus especially relevant to the subjects included in this series. As in the aforementioned studies, a large contributor to the superiority of NPWT over conventional wound care is the benefit of ongoing outpatient therapy. Ahmad et al. performed a study comparing home NPWT with expected wound care in the hospital and noted the superiority of healing in the outpatient group [11]. This benefit is lost in many of the minority populations who may be uninsured, or whose social situation prevents this home therapy. These patients are more likely to experience poor or delayed wound healing after surgical intervention when compared to the general population [12]. This limitation may be a factor playing a part in the healthcare disparity observed in access to NPWT.

As documented by the clinical courses described in this case series, we note objectively positive outcomes in our study. A limitation of this series is that we are unable to comment on the effectiveness of NPWT as it relates to the duration of the therapy relative to the general population, because of the wide range of disparity in size, location, and severity of wounds.

From our review of the literature, we did not note any specific research on NPWT in patients with limited access to healthcare resources. The authors draw attention to this case series to illustrate the profound impact that NPWT has on a diverse array of severe, complex wounds.

The future directions for research in the form of quality improvement initiatives and large prospective studies in the minority cohort can assist in addressing the healthcare disparity faced in this setting.

The authors acknowledge that these outcomes and hospital courses may not be applicable to all members of the general public; however, this series intends to identify the courses of specific patients in a minority population. The authors also acknowledge that this is a case series with a relatively small sample size, necessitating the need for larger studies on healthcare disparities and their implications on wound healing to implement successful quality improvement measures.

## Conclusions

NPWT has been used for decades in the management and treatment of complex wounds, with debated wound outcomes and cost benefits within the literature. Existing literature does not carefully examine minority populations or those without insurance, and these patients often lose a major benefit of NPWT. This benefit loss is largely because of limited options for outpatient use, which further begets the healthcare disparity as it relates to complex wound care. This case series identifies several complex wounds of different locations and etiologies within this population, with favorable outcomes after NPWT, establishing a platform for further studies within minority populations to establish more conclusive outcome data.
